# Infrared thermal imaging for detection of peripheral vascular disorders

**DOI:** 10.4103/0971-6203.48720

**Published:** 2009

**Authors:** S. Bagavathiappan, T. Saravanan, John Philip, T. Jayakumar, Baldev Raj, R. Karunanithi, T. M. R. Panicker, M. Paul Korath, K. Jagadeesan

**Affiliations:** SMARTS, NDED, Indira Gandhi Centre for Atomic Research, Kalpakkam - 603102, T.N, India; 1 KJ Hospital Research and Postgraduate Centre, 182. Poonamallee High Road, Chennai - 600 084, T.N., India

**Keywords:** Blood flow, infrared thermal imaging, noninvasive, temperature, vascular

## Abstract

Body temperature is a very useful parameter for diagnosing diseases. There is a definite correlation between body temperature and diseases. We have used Infrared Thermography to study noninvasive diagnosis of peripheral vascular diseases. Temperature gradients are observed in the affected regions of patients with vascular disorders, which indicate abnormal blood flow in the affected region. Thermal imaging results are well correlated with the clinical findings. Certain areas on the affected limbs show increased temperature profiles, probably due to inflammation and underlying venous flow changes. In general the temperature contrast in the affected regions is about 0.7 to 1° C above the normal regions, due to sluggish blood circulation. The results suggest that the thermal imaging technique is an effective technique for detecting small temperature changes in the human body due to vascular disorders.

## Introduction

The correlation of body temperature and diseases has been known for centuries, but in recent years, due to advent of new technologies, skin temperature has been used as a convenient and effective diagnostic tool to detect diseases.[1–2] Human body temperature has been recorded with thermocouples, thermistors, and thermopiles, for almost 60 years, and these sensors are very large in size, slow in response, and difficult to attach to the skin.[3] The first documented application of thermography was a method of research for early preclinical diagnosis of breast cancer in the year 1956.[4] Infrared thermography or infrared imaging or thermal imaging is a non-contact tool, which maps the surface temperature of a body or an object and it has a wide range of applications starting from condition monitoring in industries to medical imaging.[5–9] Medical infrared thermal imaging has been used to study the flow of blood, the detection of breast cancer, and muscular performance of the human body.[10,11] Thermal images have been used to quantify sensitive changes in skin temperature in relation to certain diseases.[12] Blood flow can be assessed by many methods including the washout technique, laser Doppler flowmetry,[13] and medical infrared thermal imaging.[14] Of these, infrared thermography has the advantages of being noninvasive,[15] fast, reliable, with non-contact, capable of producing multiple recordings at short time intervals, and absolutely safe for patients and doctors. In all these studies, only the relative and not absolute temperatures are significant and the relative temperatures have to be measured at many points on the skin, and in this sense, the Infrared (IR) sensing device has many advantages over conventional devices.[16,17] IR radiation covers a wavelength that ranges from 0.75μm to 1000μm, among which the human body emissions that are traditionally measured for diagnostic purposes occupy a narrow band of wavelengths ranging from 8μm to 12μm. This region is also referred as the long wave IR (LWIR) or body infrared rays. Another terminology that is widely used in medical IR imaging is thermal infrared (TIR), where the wavelength is beyond 1.4μm. Within this region, the infrared emission is primarily heat or thermal radiation. The image generated by TIR imaging is referred to as thermogram. The near infrared (NIR) region occupies wavelengths between 0.75μm and 1.4μm. Although the NIR and mid-wave IR (MWIR) regions are not traditionally used in human body screening, the new generation detectors enable the use of multispectral imaging in medicine, in which these regions are observed in different diagnostic cases.[18,19] The fundamental equations that link the absolute temperature of the object with the intensity and wavelength of the emitted radiation are given by the Planck's, Stefan Boltzmann, and Wein's Displacement law.[20] The energy radiation after Stefan Boltzman law is W = ɛ σ T^4^, where ɛ is the emissivity and T the absolute temperature. The emissivity (ɛ) of a material is the ratio of energy radiated by a particular material to the energy radiated by a black body at the same temperature. It is a measure of a material's ability to radiate absorbed energy. A true black body would have an emissivity value of unity (ɛ = 1), while any real object would have ɛ < 1. Emissivity depends on factors such as, temperature, emission angle, and wavelength. For a black body the total heat energy radiation is proportional to T^4^. A perfect black body is a perfect emitter and a perfect absorber for all wave length energies radiated, depending on the temperature of the material. Human skin keeps the body temperatures normally at 37°C. When the skin is in cooler surroundings, it cools down, emitting heat. Similarly when skin is in warmer surroundings, it absorbs heat making the body adjust itself by sweating, to keep the temperature at 37°C. In both situations, therefore, the skin acts like a black body with emissivity of 0.98, as observed. It has been shown that the emissivity of skin (black, white, burnt, male, and female) independent of the wavelength and its value is close to 0.98.[21–23] Therefore, human beings can be treated as true black bodies. The infrared radiations from the object are converted using a suitable IR detector and displayed as color or black and white image. The colors are simply a visual aid to show the temperature differences at different regions in each image.[24] Medical infrared diagnostics uses the fact that many pathological processes in the human organs manifest themselves as local changes in heat production and also as changes in the blood flow pattern of the affected organs or tissues. Infrared thermography involves recording a sequence of thermograms at several stationary positions of the human being, inspected in his natural condition. Focal plane array (FPA) based systems are more efficient for medical applications than systems previously using single element detectors.[25–27] In clinical diagnostics infrared imaging is used as a physiological test that measures the subtle physiological changes that might be caused by many conditions, e.g., contusions, fractures, burns, carcinomas, lymphomas, melanomas, prostate cancer, dermatological diseases, rheumatoid arthritis, diabetes mellitus and associated pathology, deep venous thrombosis (DVT), liver disease, bacterial infections, etc. These conditions are commonly associated with regional vasodilation, hyperthermia, hyperperfusion, hypermetabolism, and hypervascularization[18] which generate a higher-temperature heat source. The heat emanating onto the surface from the heat source and the surrounding blood flow can be quantified by using the Pennes' bio-heat equation, as follows,

(1) $$\begin{array}{c} {\mathrm{k\Delta}}^{2}–c_{b}w_{b}(T–T_{a})+q_{m}=0 \end{array}$$

Where k is the conductivity, q_m_ is volumetric metabolic rate of tissue, is the product of the specific heat capacity and the mass flow rate of blood units per volume of tissue, T is the unknown tissue temperature, and T_a_ is the arterial temperature.[21]

## Materials and Methods

The patients were allowed to rest in a room where relative humidity and room temperature were controlled (to achieve equilibration body temperature with the ambient temperature). No parts of the patient were in contact with any hot or cold sources. Only a minimum number of persons were allowed inside the room. The patients were kept away from air convection sources. These precautions had been taken to minimize the variables that might influence temperature measurement.

The main objective in the preparation of the above protocol was to ensure all the variables that might have influence during thermal image were fixed. The patient was thoroughly examined by a team of doctors and a clinical report was recorded. Patients undergoing examination by thermal imaging were disrobed in the affected region for 15 minutes, in the room. A wall–mounted, air-conditioning unit provided the required temperature inside the room. The infrared thermal camera was positioned 1 m away from the affected portion of the patients and healthy volunteers. Standard views were taken with the camera mounted on a tripod stand. The regions of interest were the anterior, posterior, and lateral views. The same views of the corresponding contra-lateral region of the patient and of normal controls were also taken. The same region was continuously monitored on a color display unit with pseudo color, making temperature changes easily discernible.

Thermal imaging of the patients was carried out using the Thermovision-550 system. This is a compact lightweight focal plane array based system with a temperature resolution of 0.1K. A high-resolution color image is provided in real time, which can be viewed on a miniature screen provided with the system or by using an external monitor. The image is captured and stored in the removable PC-card. The surface temperature profiles of the patients are recorded and later analyzed using the IRWIN software. The thermal profile of the area of examination is compared with the counterpart region of the same subject and the same region of a healthy volunteer. Using the spot meter, area, and profiling tools, the change in temperature in the region of interest is determined.

## Results and Discussions

### Case 1

A 28-year-old male, with a history of pain in the left lower limb, which was getting aggravated on prolonged standing, was examined using thermal imaging. He had varicosity of the long saphenous system of the left lower limb. The patient was suffering from complications of varicosity for the past one year. He was using crepe bandages.

The patient was febrile and comfortable at rest and was not a smoker or user of alcohol. The respiratory system (RS), cardiovascular system (CVS), central nervous system (CNS), and per abdominal examinations were normal. Local examination of the lower limbs showed dilated veins present in the dorsal aspect of the foot, extending up to the lower one-third of the leg on the right lower limb. There were dilated tortuous veins in the dorsum of the foot in the left lower limb. The radial pulse, carotid pulse, dorsalis pedis, and posterior tibial pulse were normal.

**Figure 1:**
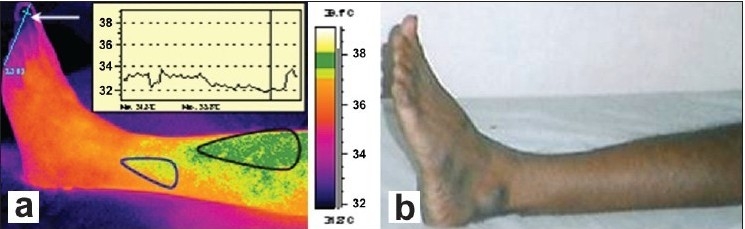
(a) Isothermal image and (b) Photograph of the affected patient's leg

Figure 1a and 1b show the thermal image and photograph of the affected patient's left leg. The line profile inset in Figure 1a shows the temperature profile along the toe tips. From the thermal image shown in Figure 1a, it can be clearly seen that a lower temperature is noted at the distal portion (indicated by white arrow in Figure 1a). This is probably due to sluggish blood circulation in the toes and venous drainage being inadequate due to the varicosity. In the patient, the area outlined by a black line, i.e., the demarcated dark-green patches, and a blue line, i.e., the demarcated pale-green color patch [Figure 1a] show abnormal temperatures compared to the temperature of the surrounding area of the same patient's leg and to that of a normal person's leg. The temperature in these marked regions is, on an average, 0.7 to 1°C above the normal regions. The abnormal temperature is due to varicose veins, with probable mild inflammation, which was not evident on clinical examination. The human body creates heat through the metabolic activity, which is the basic reaction of life. The blood in the near-surface veins, heats the surface more than the normal veins and arteries. Localized elevated temperatures are easier to discern when the person is in a cool room for at least 20 minutes. A uniform temperature can be seen in the leg of a normal person.

### Case 2

A 31-year-old male, who has a history of swelling in both the lower limbs on prolonged standing, for five years, had recurrent ulceration over the left lateral malleolus, associated with pain and discharge of pus. The patient underwent treatment and surgery four years back, for the same complaint. The RS, CVS, CNS, and per abdominal examinations were normal. Local examination of the left lower limb showed tortuous dilated veins, recurrent healing ulcers on the left lateral malleolus, ulcers covered with slough and pus discharge. Old healed scars were about 8 × 1 cm in length, present in the medial aspect of the lower limb. In the right lower limb, dilated tortuous veins, mild edema over the right ankle joint, and also old healed scars were noticed. The palpable arterial pulse was normal. The patient had systemic hypertension noted six months ago and he was under medication for the same.

**Figure 2:**
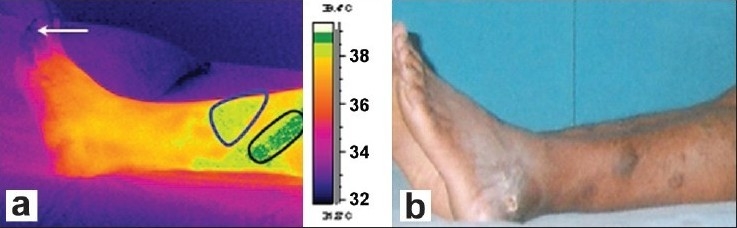
(a) Isothermal image and (b) Photograph of the affected patient's leg (Dorsal view)

Figure 2a and 2b show the dorsal thermal images and photograph of the affected patient's left leg. Clinically detected areas with varicosity show up as areas of increased warmth in the thermal images. From the thermal images, the warm areas are noted on the lateral side of the left leg as well, an unusual finding, because most patients have varicosity located only on the medial side of the leg. The distal region near the toes seems to be dark or with lower temperature due to the poor perfusion of blood (indicated by a white arrow in Figure 2a, and is attributed to stasis of circulation due to varicosity.

Areas outlined by black lines, i.e., the demarcated dark-green patch and blue line, i.e., the demarcated pale-green color patch in Figure 2a show abnormal temperature compared to the temperature of the normal person's leg, for the same region. The demarcated area in the thermal image shows a higher temperature due to the tortuous venous carrying warm blood at a sluggish speed when compared to normal venous drainage and probable mild inflammation in those areas. The temperature changes as noted on the patient are not seen in the leg of the normal person.

### Case 3

A 48-year-old male has had pain in the left leg (calf muscle) for the past two years. The pain has been severe for the past six months. He has had a history of pain aggravation on walking and pain being relieved by rest. On prolonged standing the pain increased. The RS, CVS, CNS, and per abdominal examinations were normal. The upper limb pulses were felt normally in the right and left lower limbs, the dorsalis pedis was normal in the right, with feeble low volume in the left lower limb, and the posterior tibial pulse was normal on the right, with low volume on the left. The patient is an occasional smoker and user of alcohol. There was an injury in the left big toe eight months ago. He had a nonhealing ulcer on the left great toe and gangrenous tissue was found on the great toe.

From the thermal images, the left leg medial view of the patient shows elevated temperatures because of thrombosis, a condition marked by blood clotting within the blood vessels. This disease may be potentially life threatening if dislodgment of the thrombus results in pulmonary embolism. It may be burger disease because of arterial insufficiency. It is an arterial obstruction. The clinically recorded information shows severe pain in the calf muscle, the area represented in the thermal image as a warm area shows abnormal temperature compared to the temperature of the normal person's leg, for the same region. These temperature changes are not seen in the thermal image of the normal person's left leg.

### Case 4

A 40 year-old-male had a swelling in the little finger of the left hand that was two months old. The swelling was present with a pricking type of pain and pus discharge from the left ring, middle, and index fingers. Pain was radiating from the left hand and forearm to the left chest and distal phalanges. The RS, CVS, CNS, and per abdominal examinations were normal. Local examination of the patient's right upper limb was normal. The left upper limb on inspection showed gangrenous swelling with inflammation in the left little, ring, middle, and index fingers. There was purulent discharge from the nail beds that had a foul smell. There was hyperpigmentation present in the left palm. The patient was a smoker for the past 10 years (10 – 15 beedis per day), and an occasional user of alcohol. He has no history of any surgery in the past. Due to pain he was unable to sleep and has had a reduced appetite.

From the thermal images it is clearly seen that the temperature of the finger tips of the left hand is cooler than the normal body temperature, which may be attributed to vascular insufficiency. These abnormalities are due to ischemic necrosis (death of tissue affected by local injury due to loss of blood supply) of the distal phalanges. It can be seen that the temperature increase in the affected person's hand was almost 1.5°C compared to the normal hand.

## Conclusions

Thermal imaging has been successfully used for medical diagnosis of vascular disorders. The temperature in the affected regions of patients with vascular disorders was low in the extremities due to obstructed arteries. However, in some areas it showed 0.7 to 1°C higher temperature than the normal areas due to inflammation and venous flow alteration. In general the thermal image findings were in good agreement with the clinical findings. However, the areas showing higher temperature contrast were noted not to be obvious in the clinical examination. This study demonstrates the usefulness of thermal imaging for medical diagnostics, with high reliability.
